# Comparison of trends in *Clostridioides difficile* infections in hospitalised patients during the first and second waves of the COVID-19 pandemic: A retrospective sentinel surveillance study

**DOI:** 10.1016/j.lanepe.2022.100424

**Published:** 2022-06-28

**Authors:** Karuna E.W. Vendrik, Amoe Baktash, Jelle J. Goeman, Céline Harmanus, Daan W. Notermans, Sabine C. de Greeff, Ed J. Kuijper, C. difficile surveillance study group

**Affiliations:** a Centre for Infectious Disease Control, National Institute for Public Health and the Environment (Rijksinstituut voor Volksgezondheid en Milieu, RIVM), Bilthoven, the Netherlands; b Department of Medical Microbiology, Leiden University Medical Center, Leiden, the Netherlands; c Department of Biomedical Data Sciences, Leiden University Medical Center, Leiden, the Netherlands; d Department of Medical Microbiology, Amsterdam University Medical Centers, location Academic Medical Center, Amsterdam, the Netherlands

**Keywords:** *Clostridioides difficile*, CDI, COVID-19, SARS-CoV-2, Epidemiology

## Abstract

**Background:**

During the COVID-19 pandemic, several factors, such as improved hand hygiene, social distancing, and restricted hospital referral, may have had an influence on the epidemiology of *Clostridioides difficile* infections (CDI).

**Methods:**

The annual CDI incidence rate of nine hospitals participating in the Dutch sentinel CI surveillance with complete data was compared between 2020 and the previous five surveillance years. Trends in characteristics of hospitalised CDI patients in 21–24 participating hospitals were compared between the first (March 13–May 12, 2020) or second Dutch COVID-19 wave (September 17, 2020-January 1, 2021) and the same calendar periods in 2015 through 2019. All analyses were adjusted for trend changes over time.

**Findings:**

The annual CDI incidence rate in 2020 was lower compared to previous years. During the second wave, the percentage of CDI patients with severe CDI was higher compared to earlier (25·8% in 2020 vs 17·9% in 2015-2019 (RR 1·6; 95%CI 1·1-2·3)). After adjustment for delayed *C. difficile* diagnostics (≥8 days from start symptoms), the increase disappeared. Delayed *C. difficile* diagnostics was indeed more common during the second wave (RR 1·7; 95%CI 1·1-2·6), but only for community-onset CDI (CO-CDI).

**Interpretation:**

This study shows that a higher percentage of severe CDI cases was observed during the second COVID-19 wave. This may partially be caused by delayed diagnostics, potentially due to decreased visits to a physician or restricted hospital referral for CO-CDI patients.

**Funding:**

Dutch ministry of Health.

**Research in context**

**Evidence before this study**

During the coronavirus disease 2019 (COVID-19) pandemic, several factors, such as improved hand hygiene, social distancing, and restricted hospital referral, may have had an influence on the epidemiology of *Clostridioides difficile* infections (CDI). On the 12th of January 2022, PubMed was searched for studies reporting on the epidemiology of *Clostridioides difficile* infections during the COVID-19 pandemic. The search strategy is described in the Supplementary Methods. The search included terms on COVID-19 and *C. difficile*. This PubMed search yielded 22 relevant studies. Four studies found no change in the CDI incidence rate or the percentage of patients with CDI during the COVID-19 pandemic, five studies found a decrease, and three studies found an increase. Furthermore, one study found no change in the standardised infection ratio (SIR) and one study a decrease in the SIR. One of the 22 studies compared COVID-19 patients or patients with COVID-19 in the past history with non-COVID-19 patients in the pandemic period and concluded that the percentage of patients with CDI was similar. One study examined CDI severity among COVID-19 patients during the pandemic in comparison with a period before: they found no significant difference (45 and 40%, respectively). Two studies compared COVID-19 patients with CDI to COVID-19 patients without CDI and both found that the percentage of CDI patients recovering without complications was lower and the hospital stay was prolonged. They observed 42% of 38 patients and 35% of 40 patients with CDI that were transferred from nursing homes. No other studies have examined transfers from nursing homes. Furthermore, in these two studies 29% and 35% had severe CDI, but there was no comparison to previous years. A complicated CDI course was investigated in four studies. Sehgal *et al.* examined 21 CDI patients with COVID-19 of which two were admitted to the ICU with severe CDI and none died due to CDI. Granata *et al.* found no emergency surgery for CDI and 4.7% CDI mortality among COVID-19 patients with CDI. Marinescu *et al*. found 45% of COVID-19 patients with CDI with a complicated CDI course, including 5% CDI-mortality. Only one study compared it with the pre-pandemic period: Manea *et al.* found no emergency surgery or ICU admissions due to CDI and no CDI-mortality during the COVID-19 pandemic, compared to no emergency surgery or ICU admissions and 4% CDI-mortality in the pre-pandemic period. Apart from one hospital that found no difference in department of stay compared to earlier, no other studies examined this.

**Added value of this study**

To our knowledge, this is the first study examining several clinical and epidemiological features of CDI patients in different COVID-19 waves with a comparison to the pre-COVID-19 period. No previous study has investigated time to *C. difficile* diagnostics and provided information on molecular typing of the *C. difficile* isolates. Our results are based on a large nationwide sentinel surveillance study, whereas most published studies included data of only one hospital and had a considerably smaller sample size.

**Implications of all the available evidence**

This study described the consequences of the COVID-19 pandemic and associated measures on the CDI incidence rate and characteristics of hospitalised CDI patients in the Netherlands. The results show a higher percentage of severe CDI during the second wave, presumably related to delayed *C. difficile* diagnostics. This suggests that restricted hospital referral and/or decreases in visits of patients to a physician due to the COVID-19 pandemic may have played a role. It is important that routine diagnostics are continued, even during a pandemic.

Alt-text: Unlabelled box

## Introduction

Since the end of 2019, the world has been heavily occupied with the coronavirus disease 2019 (COVID-19) pandemic and its consequences. COVID-19 can present with several symptoms, such as loss of smell, nasal obstruction, fever, or shortness of breath. Interestingly, 19% of patients have diarrhoea.^1^ In the Netherlands, the first COVID-19 case was identified in February 2020 and since then more than 110,000 patients have been hospitalised due to COVID-19.^2^ The COVID-19 pandemic has led to substantial periodic adjustments in the routine care of hospitals. There was increased hand hygiene and social distancing, more emphasis was laid on personal protective equipment and environmental cleaning, and more patients were nursed in isolation. In the peak periods of COVID-19, referral of patients and treatments for non-COVID-19 diseases was restricted to only urgent cases, transfer of COVID-19 patients between hospitals occurred, and more patients were nursed per healthcare worker with less experienced personnel on intensive care units (ICU). Interestingly, the total use of antibiotics had decreased in 2020 compared to 2019 in both the Netherlands and in the EU/EEA overall.^3^ All these factors may have had an influence on the epidemiology of *Clostridioides difficile* infections (CDI). CDI is recognised as an important antibiotic-associated infection, which is easily transmissible by spores and occurring both in the healthcare and community setting.^4^ Symptoms range from mild diarrhoea to life-threatening toxic megacolon.

Since 2009, the Leiden University Medical Center (LUMC) harbours the Dutch national reference laboratory for *C. difficile* in collaboration with the National Institute of Public Health and the Environment (RIVM). In the Dutch sentinel CDI surveillance, patient information, clinical characteristics, and 30-day outcomes from hospitalised CDI cases that meet the CDI case definition are collected. This is provided by medical microbiology laboratory (MML) staff from local hospitals via online questionnaires based on data that are subtracted from electronic medical records. In addition, *C. difficile* isolates or stool samples are sent to the reference laboratory for PCR-ribotyping, and, in case of suspicion of an outbreak, multiple locus variable-number of tandem-repeats analysis (MLVA)^5^ is also performed.

The aim of this study was to assess whether the CDI incidence and clinical and microbiological characteristics of CDI differed during the COVID-19 pandemic compared to previous years. Molecular typing of C. difficile isolates was also included to recognize clusters and outbreaks in different COVID-19 waves with a comparison to the pre-COVID-19 period.

## Methods

### Data collection and analysis

A retrospective surveillance study was performed using the data of the Dutch national sentinel CDI surveillance programme in which all hospitalised patients with CDI in participating hospitals are registered. Clinical data derived from the online questionnaires from CDI patients and microbiological data of the associated *C. difficile* isolates included in the period from the 1^st^ of January 2015 until the 1^st^ of January 2021 were collected and analysed. The reason for *C. difficile* testing and the *C. difficile* testing methods were determined by the local hospital. To assess the presence of potential bias, *C. difficile* testing methods were compared between the different years. Hospital/laboratory data, such as the testing methods and the number of patient-days, were retrieved from the annual CDI surveillance questionnaires completed by the MML staff of the hospitals.

The applied CDI and severe CDI case definitions were similar as described before.^6^ The in- and exclusion criteria are described in the Supplementary Methods. CDI was classified as severe if one or more of the following conditions were present^6^: 1. fever (temperature of 38°C or higher) and leucocytosis (>15  ×  10^9^/L), 2. hypoalbuminemia (<20 g/L) and/or dehydration, 3. pseudomembranous colitis, and/or 4. bloody diarrhoea. Furthermore, a complicated CDI course was defined as the need for an emergency surgical procedure due to CDI, admission to an ICU due to CDI, and/or mortality due to or partly due to CDI within 30 days after CDI diagnosis. A recurrence was defined as a CDI episode occurring within eight weeks after onset of a previous CDI episode with clinical symptoms and a positive CDI test. Time to *C. difficile* diagnostics was measured by the difference between start of symptoms and sample date: rapid diagnostics (< 3 days), moderately rapid (3-8 days), and delayed diagnostics (≥8 days). Community-onset of symptoms (CO-CDI) was defined as start of symptoms outside of a health care facility. Age was divided into four categories: below 18, 18–65, 65–85 and above 85 years old.

### PCR-ribotyping and multiple locus variable-number of tandem repeats analysis (MLVA)

All *C. difficile* isolates, received or cultured by the national reference laboratory, were characterised with PCR ribotyping. PCR ribotyping was performed using capillary gel electrophoresis (3500xL Genetic Analyser of Applied Biosystems, USA).^7^ To determine the genetic relatedness of strains with the same ribotype, MLVA was performed as described previously.^5^ A minimum spanning tree was constructed to determine the genetic distance between isolates, based on the number of differing loci and the summed tandem repeat difference (STRD) using BioNumerics version 7.6.3 (Applied Maths). Isolates belonged to a clonal complex or genetic cluster when there was an STRD ≤2.^8^ The five most common ribotypes (RTs) among all ribotyped isolates in 2020 were assessed and the percentage of these ribotypes among all ribotypes were compared between 2020 and 2015 through 2019. MLVA was performed on stored isolates with ribotypes that were more or less common in 2020 compared to previous years. These isolates were from the whole pandemic period and the same calendar period in 2015 through 2019 or, for RT014, for the pandemic period and 2019 only due to the large number of isolates.

### Data presentation

**Figure 1:**
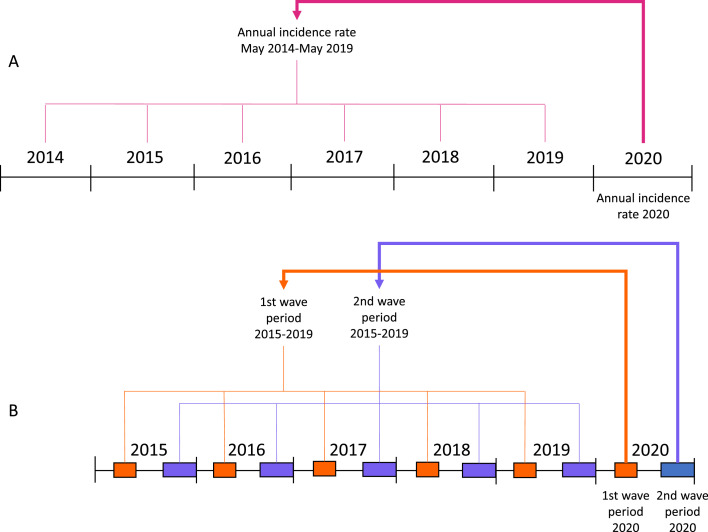
**Overview of the performed analyses in this study.** A: Comparison of the annual incidence rates of 2020 with the previous five surveillance years (year-level). B: Comparison of the percentages of CDI patients with certain characteristics in the wave periods in 2020 with that of CDI patients in the same calendar periods (wave periods) in 2015 through 2019 (patient-level).

We performed two types of analyses: comparison of incidence rates (on year level) and comparison of categorical CDI characteristics variables (on patient level). For all analyses, we compare 2020 with the previous five (surveillance) years with a Poisson regression analysis with a robust variance estimator^9^, ^10^, ^11^, ^12^ and we correct for trend changes over time by adding year (2015-2020) as covariate. Although we did not find evidence for overdispersion, we used the robust variance estimator to correct for potential overdispersion (Generalised Estimating Equations approach).^10^ The two types of analyses are explained in Figure 1.

For the comparison of incidence rates (on year level), we calculated the (mean) annual CDI incidence rate per 10,000 patient-days of all admitted patients for 2020 (1^st^ of Jan through 31^st^ of Dec) and the previous five surveillance years (May 2014 through April 2019; before 2020, surveillance incidence data was collected from May-May instead of Jan-Jan), as the number of CDI cases divided by the number of patient-days in these periods. For this calculation, data were only used from hospitals for which both the number of CDI cases and the number of patient-days were available, meaning that the data from the numerator and the denominator are from the same hospitals. Furthermore, hospitals with missing data or with varying numbers of hospital locations in 2020 or the previous five surveillance years were excluded from this analysis. Annual incidence rates were compared using multivariable Poisson regression analysis and trend-adjusted incidence rate ratios (IRR) with 95% CI were calculated. We compared the annual incidence rates of 2020 with the mean annual incidence rate of the previous five surveillance years (numeric dependent variable: number of cases per year, binary independent variable: 2020: yes/ 2015–2019: no, covariate: year (2015–2020), offset: ln(patient-days)). Besides year, no other covariates were added in this analysis. This is visualised in Figure 1A.

For the comparison of categorical CDI characteristics variables (on patient level), data are presented as number (%) of CDI patients among all admitted CDI patients in a certain period. We defined three COVID-19 periods: the first COVID-19 wave (March 13, 2020 - May 12, 2020), the second COVID-19 wave (September 17, 2020 - January 1, 2021), and the interwave (period in between). The waves were assigned for periods in which the mean number of COVID-19 hospital admissions per day of the previous seven days was more than 40 (signal value of the RIVM). To account for seasonality, we compared the percentages of CDI patients with certain characteristics hospitalised in the wave periods in 2020 with that of CDI patients hospitalised in the same calendar periods (wave periods) in 2015 through 2019. This is visualised in Figure 1B. A multivariable Poisson regression analysis was used to assess the association between an independent binary time period variable (first all comparisons for the first wave period 2020 (yes) vs the same period in 2015 through 2019 (no) and then all comparisons for the second wave period 2020 (yes) vs the same period in 2015 through 2019 (no); with a single model for each comparison) and dependent binary CDI characteristics variables (% of patients with certain CDI characteristics), corrected for trend changes over time by adding year (2015-2020) as covariate. Trend-adjusted risk ratios (RR) of the CDI characteristics for these time periods with 95% CI were calculated (see Table 1). To examine whether certain variables may play a role in the causal path for the association between time period and the percentage of patients with severe CDI, relevant covariates were added one by one in several multivariable Poisson regression analyses (on patient level) with period as binary independent variable, CDI severity as binary dependent variable and year as other covariate. In case the RR changed considerably compared to the analysis without this covariate, this suggested a role in the causal path and then the resulting mediation- and trend-adjusted RR for severe CDI with 95% CI was indicated. This RR represents the mean ratio of the risks of severe CDI in period 1 (first/second wave 2020) compared to the baseline risk in period 2 (same period in 2015 through 2019) for the different categories of the added covariates.

**Table 1:** Characteristics of CDI patients during the first and second wave periods in 2020, compared to the wave periods in 2015–2019.

		2015-2019			2020			2020 compared to 2015-2019	
		n	Total	%	n	Total	%	Risk ratio	95% CI
			P1 =832			P1=93			
			P2=1332			P2=228			
**Age categories**	1st wave period								
	*Below 18*	30	832	3·6%	2	93	2·2%	0·3	0·1–1·5
	*18-65*	254	832	30·5%	40	93	43·0%	1·3	1·0–1·9
	*65-85*	444	832	53·4%	35	93	37·6%	0·7*	0·5–0·9
	*Above 85*	104	832	12·5%	16	93	17·2%	2·0*	1·0–3·8
	2nd wave period								
	*Below 18*	31	1332	2·3%	2	228	0·9%	0·4	0·1–1·7
	*18-65*	466	1332	35·0%	73	228	32·0%	0·9	0·7–1·1
	*65-85*	658	1332	49·4%	121	228	53·1%	1·2	1·0–1·4
	*Above 85*	177	1332	13·3%	32	228	14·0%	0·9	0·6–1·4
**Sex (% female)**	1st wave period	408	832	49·0%	44	93	47·3%	1·1	0·8–1·4
	2nd wave period	684	1330	51·4%	123	228	53·9%	1·0	0·8–1·2
**Severe CDI**$	1st wave period	153	793	19·3%	14	93	15·1%	0·9	0·5–1·7
	2nd wave period	232	1293	17·9%	58	225	25·8%	1·6*	1·1–2·3
**Complicated**	1st wave period		832	2.9%		93	1.5%	0·9	0·1–8·9
**CDI course**∼#	2nd wave period		1332	2.6%		228	1.8%	1·8	0·4–9·1
**All-cause**	1st wave period		832	10.5%		93	14.4%	1·3	0·6–2·6
**mortality**£	2nd wave period		1332	10.9%		228	11.2%	1 ·1	0·7–1·9
**CDI mortality**#	1st wave period		832	2.1%		93	0.4%		
	2nd wave period		1332	1.9%		228	1.3%	1·4	0·2–10·1
**Recurrent CDI**	1st wave period	116	531	21·8%	9	60	15·0%	0·8	0·4–1·7
	2nd wave period	154	848	18·2%	20	136	14·7%	1·1	0·7–1·9
**Community-**	1st wave period	364	831	43·8%	46	92	50·0%	1·0	0·7–1·3
**onset**	2nd wave period	571	1330	42·9%	97	228	42·5%	0·9	0·7–1·1
**Time to *C.difficile***	1st wave period								
**diagnostics**	*0-2 days*	415	661	62·8%	44	80	55·0%	0·9	0·7–1·2
	*3-8 days*	134	661	20·3%	18	80	22·5%	0·9	0·6–1·6
	*8 or more days*	112	661	16·9%	18	80	22·5%	1·4	0·8–2·5
	2nd wave period								
	*0-2 days*	676	1092	61·9%	110	189	58·2%	0·9	0·8–1·1
	*3-8 days*	250	1092	22·9%	37	189	19·6%	0·9	0·6–1·3
	*8 or more days*	166	1092	15·2%	42	189	22·2%	1·7*	1·1–2·6
**Transfer from**	1st wave period	53	825	6·4%	2	93	2·2%	0·5	0·1–2·4
**nursing homes**	2nd wave period	70	1326	5·3%	21	226	9·3%	1·6	0·8–3·0

Depicted risk ratios trend-adjusted risk ratios.

⁎ p-value <0·05 as assessed by Poisson regression analysis using a robust variance estimator.

$ Defined as: 1. pseudomembranous colitis, 2. bloody diarrhoea, 3. hypoalbuminemia and/or dehydration, and/or 4, leucocytosis and fever.

∼ Defined as: admission to the ICU due to CDI, emergency surgical procedure due to CDI, or death due to CDI or partly due to CDI.

# Missing data complemented by multiple imputations with chained equations; complete data before multiple imputations: 2015-2019: 1^st^ wave period: 736, 2^nd^ wave period: 1,196; and 2020: 1^st^ wave period: 83, 2^nd^ wave period: 189. For both the complete case analysis and for the multiple imputed datasets, there were no significant differences.

£ Missing data complemented by multiple imputations with chained equations; complete data before multiple imputations: 2015-2019: 1^st^ wave period: 739, 2^nd^ wave period: 1,205; and 2020: 1^st^ wave period: 88, 2^nd^ wave period: 198. For both the complete case analysis and for the multiple imputed datasets, there were no significant differences. Abbreviations: CDI: *Clostridioides difficile* infection, CI: confidence interval.

In most analyses, a complete case analysis was performed. The number of patients with available data per variable are mentioned. Several variables, such as severe CDI, had very little missing data (<5%) overall and for each period that was included in the comparisons (1^st^ and 2^nd^ wave 2020 and 1^st^ and 2nd wave period 2015–2019). For other variables (<30% missing data), our hypothesis is that missing data were mostly missing completely at random, since persons that filled in the clinical data and outcomes were not involved in the treatment of the patient. Furthermore, the percentages of missing data did not differ considerably between periods, which means that comparisons of the periods are valid. However, some data on complicated CDI course and (CDI) mortality from between the 2^nd^ of November 2020 until the 1^st^ of January 2021 were missing or excluded due to technical issues with absence of some answer possibilities in the web-based questionnaire (5-17% missing data per period). Data from these variables were therefore partly missing at random and multiple imputations with chained equations was performed (50 imputations using (multinominal) logistic regression and linear regression). A two-sided p-value of <0·05 was considered significantly different. IBM SPSS Statistics version 25 (IBM Corp, NY, USA) and STATA SE version 15.1 (StataCorp, College Station, TX, USA) were used for data-analysis.

### Ethics statement

For this study, data from the Dutch national CDI surveillance were used that were already collected. There were no additional data or isolates/materials collected specifically for this study and no actions were requested from patients. The data do not include personally identifiable information. Written or verbal informed consent was therefore not required. According to the Dutch Medical Research Involving Human Subjects Act (WMO) this study was considered exempt from review by an Institutional Review Board.

### Role of the funding source

The Dutch ministry of Health sponsored the *C. difficile* reference laboratory and had no role in study design, data collection, data analysis, data interpretation, writing of the report or the decision to submit the paper for publication. There was no specific grant for this study.

## Results

### Hospital characteristics

The number of hospitals that participated in the sentinel CDI surveillance, was 21 for 2020 and 22 to 24 for 2015-2019. In 2020, 12/21 (57%) hospitals applied ESCMID-recommended CDI testing algorithms,^13^ compared to a mean percentage of 49% for 2015-2019.

For nine participating hospitals with complete incidence data, the CDI incidence rate was 2·9 CDI cases per 10,000 patient-days in 2020, whereas this was higher with a rate of 3·2 in the previous five surveillance years (trend-adjusted IRR 0·8; 95%CI 0·8-0·8).

### CDI patient characteristics

**Figure 2:**
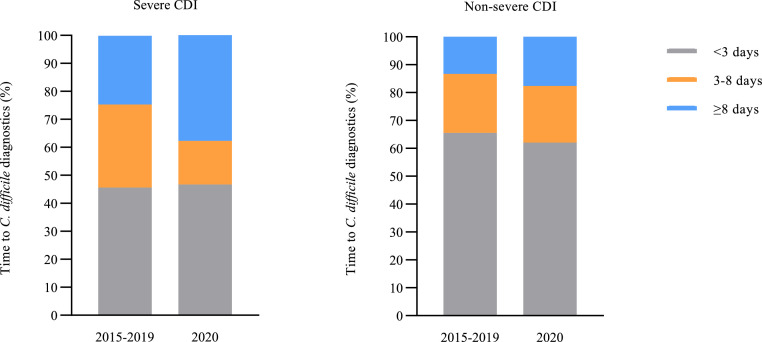
Time to *C. difficile* diagnostics in the second wave in 2020 compared to the same period in 2015 through 2019 for severe CDI and non-severe CDI patients.

Table 1 shows the numbers and percentages of CDI patients with certain characteristics among all CDI patients during the COVID-19 waves in 2020 and the same calendar period in 2015-2019. There was a different age distribution in the first wave of 2020, compared to the same calendar period in 2015-2019. A higher percentage of hospitalised patients had severe CDI during the second wave period in 2020 compared to 2015-2019 (trend-adjusted RR 1·6 (95%CI 1·1-2·3). Likewise, the percentage of patients with dehydration and/or hypoalbuminaemia was also higher with 13·3% (30/225) in 2020 versus 7·7% (100/1,293) in 2015-2019 (trend-adjusted RR 3·1; 95%CI 1·7-5·8). This higher percentage of severe CDI in the second wave of 2020 compared to 2015-2019 remained present after adjustment for transfer from nursing homes, community-onset of symptoms, age categories, or the presence of hypervirulent ribotypes (RT027 and RT078/126) in separate multivariable Poisson regression analyses. Delayed *C. difficile* diagnostics were more frequently observed during the second wave period in 2020, compared to 2015-2019 (trend-adjusted RR 1·7; 95%CI 1·1-2·6). This was only observed when CO-CDI patients were included (trend-adjusted RR 1·7 (95%CI 1·1-2·8)), not in the analysis of the group of hospital-onset CDI patients only (trend-adjusted RR 1·6 (95%CI 0·7-3·6)). When the increase in percentage of severe CDI in the second wave in 2020 was adjusted for delayed *C. difficile* diagnostics in a multivariable Poisson regression analysis, there was no significant difference in severe CDI anymore compared to 2015-2019 (mediation- and trend-adjusted RR 1·2; 95%CI 0·8-1·8), suggesting delayed *C. difficile* diagnostics was a (partial) mediator. The percentage of patients with delayed diagnostics among severe CDI patients was 37·8% during the second wave period in 2020, compared to 24·6% in the same period in 2015-2019 (Figure 2). No differences in CDI recurrences were observed in the COVID-19 waves compared with the same periods in 2015–2019.

Supplementary Figure 1 shows that the percentage of CDI patients with hospital-onset of symptoms diagnosed by general surgery physicians was significantly higher during the first wave period in 2020 with 26·8% (11/41), compared to 7·3% (30/411) in 2015-2019 (trend-adjusted RR 4·3; 95%CI 1·6-11·2). Notably, there were no CDI patients diagnosed by cardiology physicians during the first wave period in 2020, compared to 6·6% (27/411) of patients in 2015-2019. The percentage of CDI patients with hospital-onset of symptoms diagnosed by pulmonology physicians was significantly lower during the first wave period in 2020 with 2·4% (1/41), compared to 10·0% (41/411) in 2015-2019 (trend-adjusted RR 0·1; 95%CI 0·0-1·0).

### PCR-ribotyping and multiple locus variable-number of tandem repeats analysis

**Figure 3:**
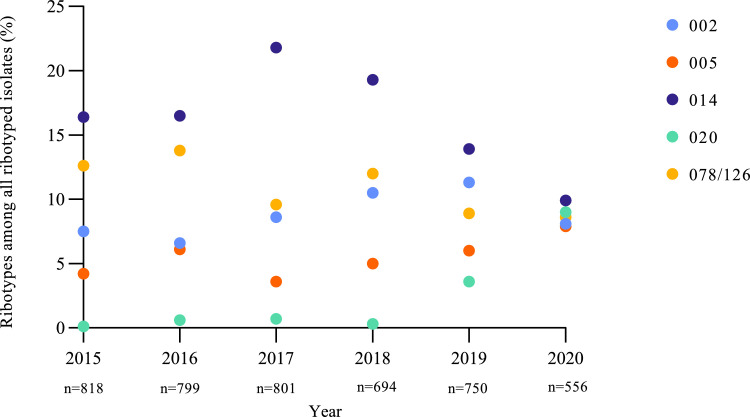
Percentage of five most common ribotypes of 2020 in time from 2015 through 2020.

Figure 3 shows the trends in percentages of the most common PCR ribotypes among all ribotyped isolates in the period from 2015 to 2020. The percentage of RT020 showed a strong increase in 2020 with 9·0% (50/556) compared to 1·1% (41/3,862) in 2015 through 2019, with a non-adjusted RR of 8·5 (95% CI 5·7-12·7). However, this effect disappeared after correction for trend changes over time (trend-adjusted RR 1·4; 95%CI 0·7-2·9). The percentage of RT014 showed a decrease compared to 2015 through 2019 from 17·6% (679/3,862) to 9·9% (55/556), with a non-adjusted RR of 0·6 (95% CI 0·4-0·7), also after correction for trends in time (trend-adjusted RR 0·6; 95% CI 0·4-0·8). The percentage of RT020 was increased during the first and second wave and interwave period in 2020, compared to the same periods in 2015–2019. However, this was also not significantly different anymore after correction for trend changes over time (Supplementary Figure 2). To analyse whether the RT020 increase was the result of a clonal spread, we performed MLVA on the 72 RT020 strains from the three periods (Supplementary Figure 3). In total, there were five small clusters with isolates from unique patients, each containing a maximum of three isolates. Two of the five clusters contained more than one isolate from 2020. As control, we also analysed RT014 strains from 2019 and 2020 and detected four small clusters with eight strains from 2019 and two strains from 2020 (Supplementary Figure 4).

## Discussion

In summary, the CDI incidence rate in 2020, predominated by the COVID-19 pandemic, was lower compared to previous years. During the second COVID-19 wave in 2020, the percentage of hospitalised CDI patients with severe CDI was higher compared to the same calendar periods in previous years. Concomitantly, the percentage of CDI patients with delayed *C. difficile* diagnostics was higher during the second wave, but only for CO-CDI. When the increase in severe CDI during the second wave was corrected for delayed *C. difficile* diagnostics, the increase was not significant anymore. In 2020, there was an increase in the percentage of RT020 infections, although this was not significant after correction for trend changes over time, accompanied with a decrease in RT014. This increase could not be explained by more clusters or spread of specific RT020 clones.

The decrease in CDI incidence rate during the COVID-19 pandemic is comparable to several other reports.^14^, ^15^, ^16^, ^17^, ^18^ It could be related to a younger patient population in the first wave with different comorbidity. Other potential explanations are improved hygiene and environmental cleaning, more protective clothing, social distancing, and less spread due to less inpatients. Furthermore, the absolute number of hospitalised CDI patients showed a decreasing trend during the first wave. This may be due to a decrease in the total number of hospitalised patients due to the restricted referral policy. Importantly, data on CDI patients that were not admitted were not available.

The decrease in the CDI incidence rate could also be caused by a decrease of the CDI testing rate, possibly due to diarrhoea as a symptom of both COVID-19 and CDI, less focus on other infectious diseases than COVID-19, or due to shortage of PCR-reagents with priority for SARS-CoV-2 tests and shortage of personnel. In the Netherlands, a shortage of PCR-reagents and personnel resulted in a temporary transition to other types of *C. difficile* diagnostics such as toxin Enzyme Immunoassay (EIA), but these tests were only allowed to perform in specific biosafety cabinets and were therefore more difficult to perform. Among 13 hospitals participating in the Dutch surveillance that provided data for both surveillance periods, 57·1 CDI tests were performed per 1,000 hospital admissions with 8·0% of positive CDI tests for May 2019-Jan 2021 and 62·3 tests with 7·6% of positive CDI tests for May 2018-May 2019. Several other studies found a reduction^19^^,^^20^ or no difference^15^^,^^21^ in the number of CDI tests and one of these studies found a decrease in the number of CDI tests per admission.^15^ However, one study found an increase in the number of CDI tests.^22^ These numbers could differ per country. Most studies found a similar percentage of positive CDI tests.^19^^,^^21^^,^^23^ The percentage of hospitals applying ESCMID-recommended CDI testing algorithms in the Netherlands increased from 46% in 2015 to 57% in 2020. The use of NAAT only (38%) or toxin EIA only (0%) in 2020 compared to the mean of 2015-2019 (42% and 5%) were both slightly lower. Use of a toxin EIA only could underestimate the CDI incidence, but NAAT could overestimate the CDI incidence.^13^ The under- and overestimation of CDI in 2015–2019 were similar. Therefore, we consider it unlikely that the CDI incidence rate in 2020 and 2015-2019 has been affected by different CDI testing methods.

During the second COVID-19 wave, the percentage of patients with severe CDI among hospitalised patients was higher compared to previous years. After adjustment for delayed *C. difficile* diagnostics, the percentage of patients with severe CDI was not significantly higher anymore. Furthermore, more frequent delayed diagnostics was only observed in CO-CDI patients. The results suggest that restricted hospital referral and/or decreases in visits to a physician before and/or during the second wave may have resulted in an increased number of severe CDI. General practitioners tend to request less *C. difficile* diagnostics compared to physicians in the hospital.^24^ However, other reasons for the delayed *C. difficile* diagnostics and increase in severe CDI cannot be excluded. Unfortunately, COVID-19 status and other comorbidities were unknown. By adding year as covariate, we corrected for trend changes over time. Unfortunately, numbers of CDI patients were too low to also add month as covariate. One other study compared the percentage of patients with severe CDI between the COVID-19 pandemic and the period before and they found no significant difference (40 and 45%).^25^ However, a different definition of severe CDI was used and there was no distinction between first and second wave. Unfortunately, data on CDI treatment, which may potentially impact CDI severity, were not available in this study. However, since CDI treatment recommendations in the Netherlands have not changed in 2015-2020 and no changes were observed in recurrences, we consider it unlikely that the observation of more severe CDI in the second COVID-19 wave was attributed to anti-CDI treatment.

During the first COVID-19 wave, there was an absolute and relative increase of CDI patients that were diagnosed by general surgery physicians. This may be caused by overcrowded internal medicine and pulmonology departments, but could not be analysed. Furthermore, there was a relative and absolute decrease in CDI patients diagnosed by cardiology physicians and pulmonology physicians. For cardiology, this may be explained by the absolute decrease of cardiology patients due to a decrease in patients that presented with cardial complaints during the COVID-19 pandemic in the Netherlands.^26^ For pulmonology, there may have been less CDI testing due to more attention towards COVID-19 symptoms or an actual lower incidence of CDI among COVID-19 patients.

The distribution of the five most common PCR ribotypes in time appeared different for 2020 compared to 2015–2019. The increase of RT020 and the decrease of RT014 appeared to have started yet in 2019. It was not caused by a substantial change in the number of clusters during the pandemic. This was observed in all hospitals. The cause of this increase remains unknown. However, the increase of RT020 was smaller in the interwave period, suggesting there were COVID-19 pandemic-associated factors involved. A limitation is the use of PCR ribotyping and MLVA to characterise a selected number of *C. difficile* isolates. Whole genome sequencing of all collected isolates could have provided more details on unidentified PCR ribotypes and transmission of strains not belonging to RT014 or RT020.

This study examined the CDI incidence and multiple CDI patient and bacterium characteristics during both the first and second COVID-19 wave compared to previous years. The generalisability of the results are difficult to estimate, since every country implemented different measures to combat the pandemic. However, this study shows the importance of examining the effect of the pandemic on CDI. The results suggest that restricted hospital referral and/or decreases in visits to a physician during the pandemic may have played a role in an increased percentage of severe CDI cases. Despite all restrictions in access of care, it is important that routine diagnostics are never postponed, even during a pandemic.

## Contributors

The *C. difficile* surveillance was conceived by EJK, SCDG, and DWN. The study was devised and supervised by EJK and SCDG. KEWV, AB, and EJK coordinated the *C. difficile* surveillance. AB collected the data for one hospital, with support of KEWV. The *C. difficile* surveillance study group collected the data for the other hospitals. AB aggregated the hospital data from all hospitals with support from KEWV. KEWV and AB accessed and verified the underlying data. KEWV performed the analyses and wrote the first draft of the manuscript. KEWV and EJK were responsible for the decision to submit the manuscript. JJG provided support for the statistical analyses. CH performed PCR ribotyping and MLVA. All authors critically reviewed the manuscript.

## Data sharing statement

After de-identification, individual patient data and/or STATA codes, that underlie the results reported in this article, will be shared from the date of publication until one year after publication, when researchers want to achieve aims of a provided methodologically sound proposal. Proposals should be directed to karunavendrik@gmail.com to gain access. Data requestors will need to sign a data access agreement. Data will then be shared via a Mendeley DOI.

## Declaration of interests

We declare that we have no conflicts of interest.

## Appendix Supplementary materials

Image, application 1

Image, application 2
