# Supplementary material for: Comparison of trends in Clostridioides difficile infections in hospitalised patients during the first and second waves of the COVID-19 pandemic: A retrospective sentinel surveillance study
Source: Lancet Reg Health Eur. 2022 Jun 28;19:100424. doi: 10.1016/j.lanepe.2022.100424 (PMC9236856; doi:10.1016/j.lanepe.2022.100424)
Supplement: Supplementary file 1 [file mmc1.docx]

**Supplementary material**

**Comparison of trends in *Clostridioides difficile* infections in hospitalised patients during the first and second waves of the COVID-19 pandemic: A retrospective sentinel surveillance study**

Karuna EW Vendrik, MD, Amoe Baktash, MD, Jelle J Goeman, PhD, Céline Harmanus, MSc, Daan W Notermans, PhD, Sabine C de Greeff, PhD, Ed J Kuijper, PhD On behalf of the *C. difficile* surveillance study group.

**Contents**

[Supplementary methods 2](#_Toc101344625)

[Search strategy 2](#_Toc101344626)

[In- and exclusion criteria 2](#_Toc101344627)

[Supplementary results 3](#_Toc101344628)

[Supplementary figures 3](#_Toc101344629)

[Supplementary Figure 1. Distribution of diagnosing specialties of CDI patients with hospital onset of symptoms during the different periods. 3](#_Toc101344630)

[Supplementary Figure 2. Percentage of ribotype 020 among all ribotyped isolates during the COVID-19 pandemic wave periods and the interwave period in 2020, compared to the same periods in 2015 through 2019. 4](#_Toc101344631)

[Supplementary Figure 3. Multiple Locus Variable-Number of Tandem-Repeats Analysis (MLVA) of ribotype 020 strains collected during the COVID-19 waves and interwave period in 2020 and the same calendar periods in 2015 through 2019. 5](#_Toc101344632)

[Supplementary Figure 4. Multiple Locus Variable-Number of Tandem-Repeats Analysis (MLVA) of ribotype 014 strains collected during the COVID-19 waves and interwave period in 2020 and the same calendar periods in 2019. 6](#_Toc101344633)

# Supplementary methods

## Search strategy

Pubmed was searched on the 12th of January 2022 with the following search strategy:

("COVID-19"[tiab] OR "COVID 19"[tiab] OR "SARS-CoV-2"[tiab] OR "SARS CoV 2"[tiab] OR "2019-nCoV"[tiab] OR "COVID-19"[mesh]) AND ("difficile"[tiab] OR "Clostridioides difficile"[mesh])

## In- and exclusion criteria

*Inclusion criteria:*

Every patient that is admitted to a participating hospital with a clinical suspicion of *C. difficile* infection and that meets these two criteria:

- Clinically diagnosed diarrhoea or toxic megacolon.
- a positive test for the presence of *C. difficile* toxins in faeces or detection of a toxin-producing *C. difficile* strain via culture/PCR or a pseudomembranous colitis as observed during endoscopy, surgery or histopathology.

*Exclusion criteria:*

- Every additional positive *C. difficile* sample of the same patient within two weeks after the previous *C. difficile* sample.
- Every sample of the same diarrhoeal episode.
- Samples of persons that are not admitted to the hospital.
- Patients that are younger than two years old.

# Supplementary results

# Supplementary figures


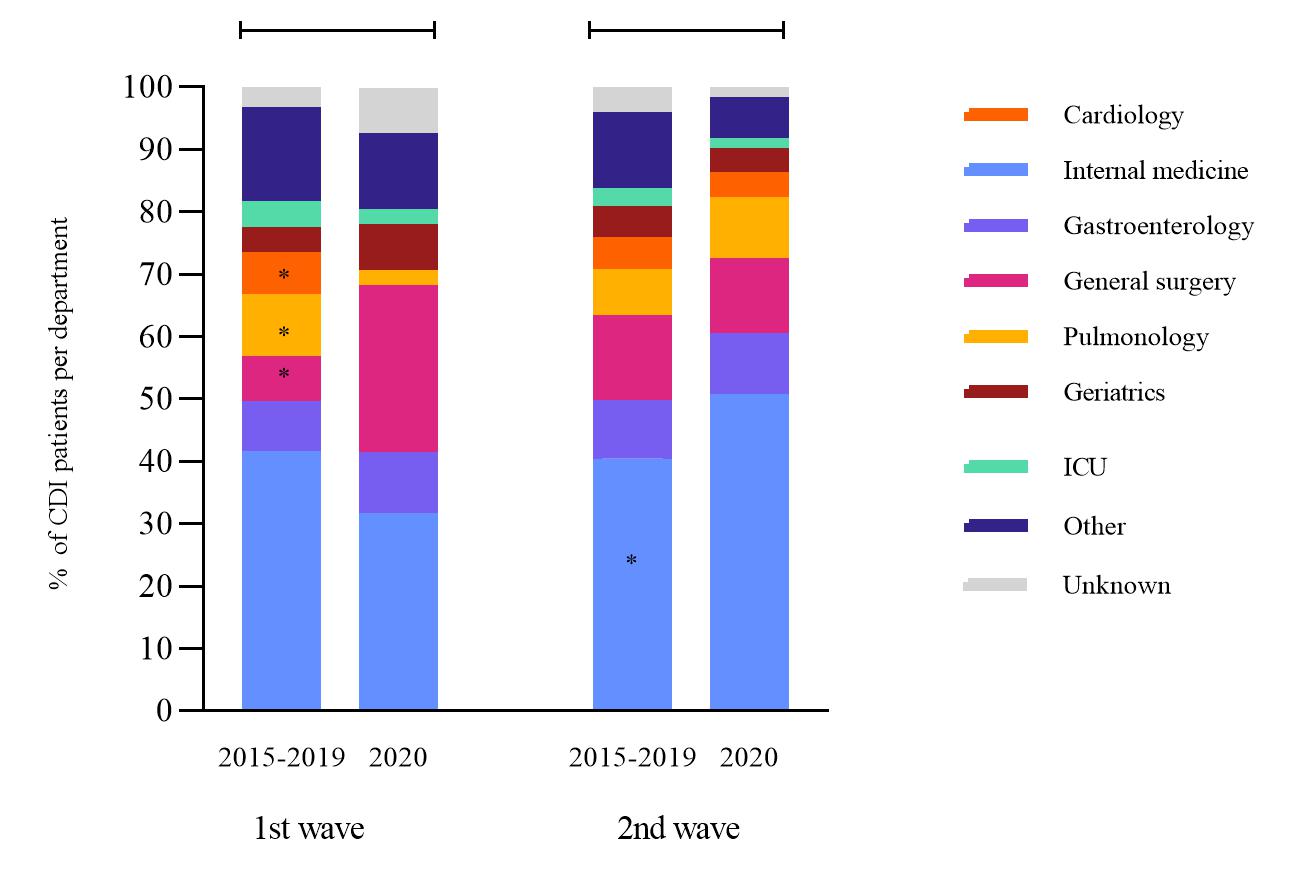


## Supplementary Figure 1. Distribution of diagnosing specialties of CDI patients with hospital onset of symptoms during the different periods.

Significant differences as assessed by multivariable Poisson regression analysis with a robust variance estimator between percentages of 2020 compared to 2015-2019 (with correction for trend changes over time) were indicated by a black star and brackets. Abbreviations: CDI: *Clostridioides difficile* infections, ICU: intensive care unit.


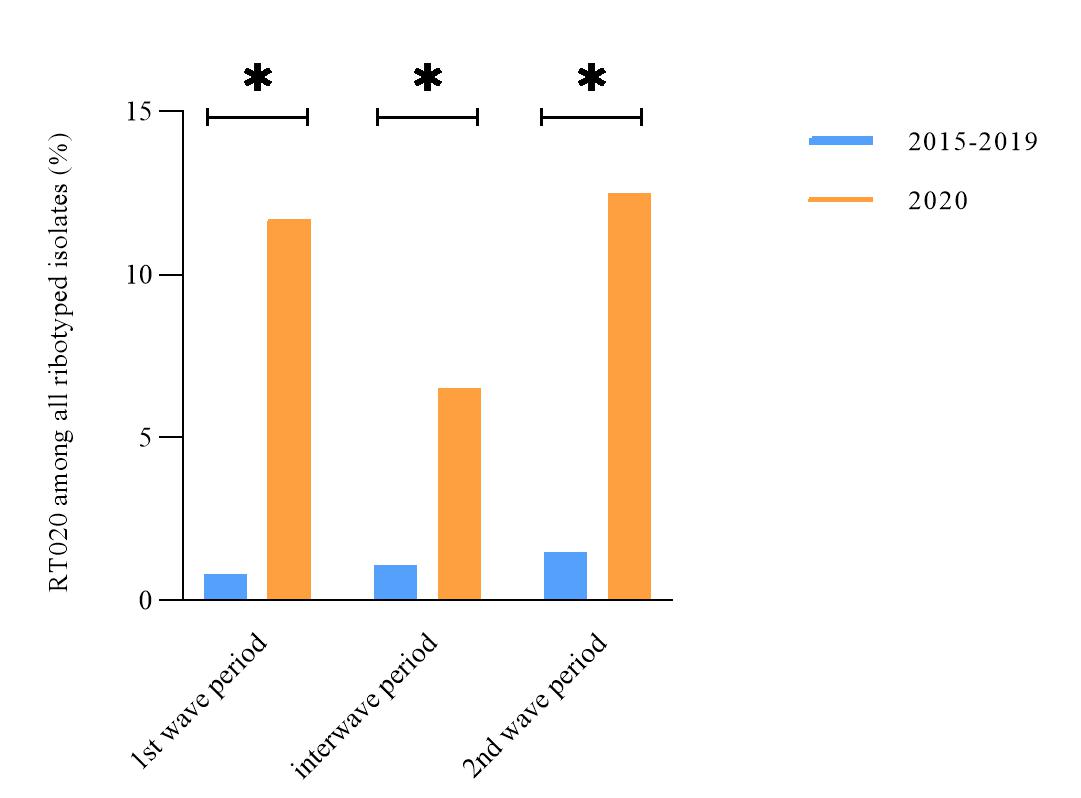


## Supplementary Figure 2. Percentage of ribotype 020 among all ribotyped isolates during the COVID-19 pandemic wave periods and the interwave period in 2020, compared to the same periods in 2015 through 2019.

Significant differences as assessed by multivariable Poisson regression analysis between percentages of 2020 compared to 2015-2019, without correction for trend changes over time, were indicated by brackets and a blue star. There were no significant differences after correction for trend changes over time.


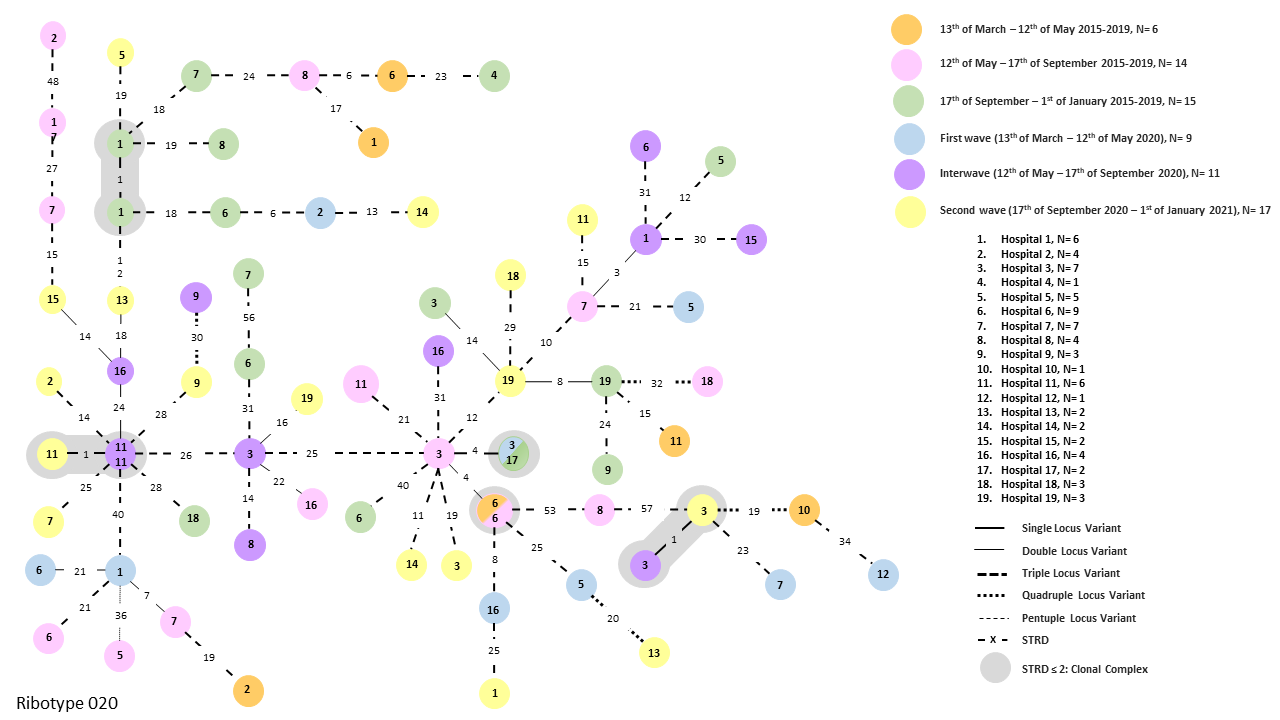


## Supplementary Figure 3. ****Multiple Locus Variable****-Number of Tandem-Repeats ****Analysis**** (MLVA) of ribotype 020 strains collected during the COVID-19 waves and interwave period in 2020 and the same calendar periods in 2015 through 2019.

Abbreviations: STRD: summed tandem repeat difference. Two RT020 isolates, one from hospital 7 and one from hospital 17, were not available anymore for MLVA.


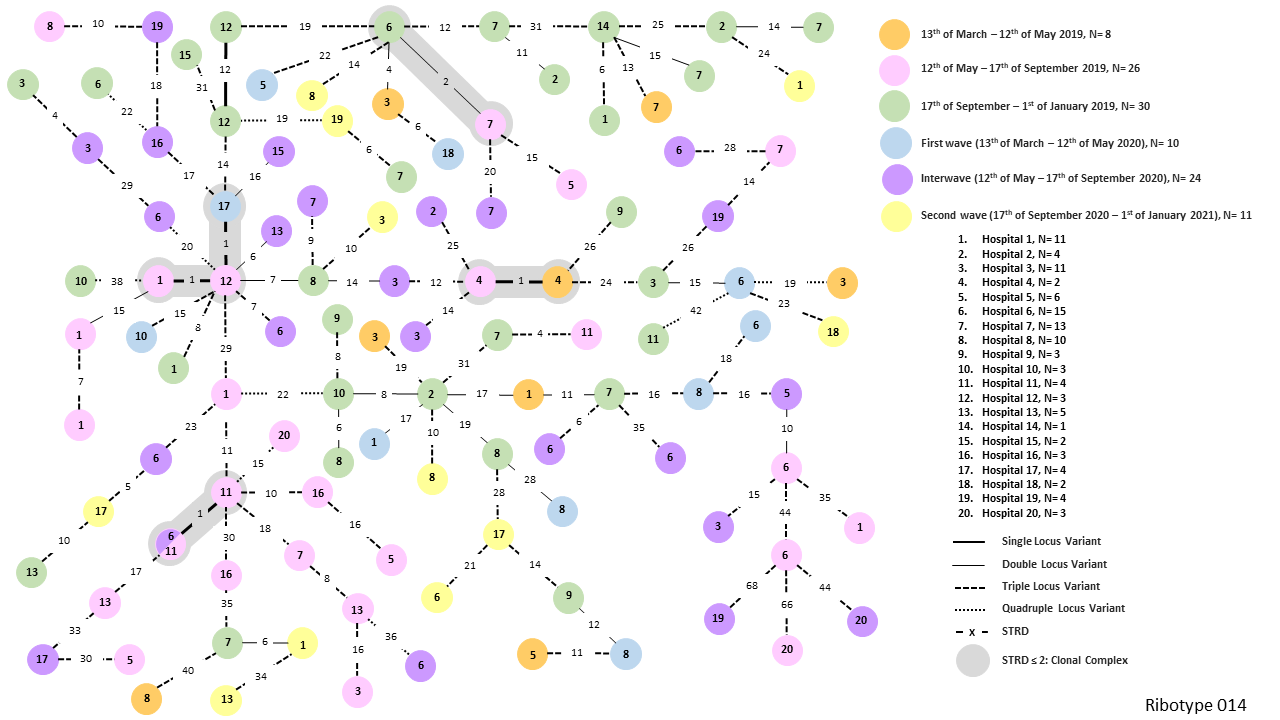


## Supplementary Figure 4. ****Multiple Locus Variable****-Number of Tandem-Repeats ****Analysis**** (MLVA) of ribotype 014 strains collected during the COVID-19 waves and interwave period in 2020 and the same calendar periods in 2019.

Abbreviations: STRD: summed tandem repeat difference.
