# Supplementary material for: Comparison of trends in Clostridioides difficile infections in hospitalised patients during the first and second waves of the COVID-19 pandemic: A retrospective sentinel surveillance study
Source: Lancet Reg Health Eur. 2022 Jun 28;19:100424. doi: 10.1016/j.lanepe.2022.100424 (PMC9236856; doi:10.1016/j.lanepe.2022.100424)
Supplement: Supplementary file 2 [file mmc2.docx]

***C. difficile* surveillance study group:**

| **Surname** | **First and middle names/initials** |
| --- | --- |
| Veenemans | Jacobien |
| Visser | Caroline |
| van Dessel | Helke |
| Weterings | Veronica |
| da Silva | Júlia Maria |
| Vlek | Anneloes |
| Vos | Margreet C. |
| van Burgel | Nathalie |
| Boot | Erik |
| Sturm | Patrick D. |
| Buijtels | Patricia |
| Jansen | Rogier |
| van der Vorm | Eric |
| Bosboom | Ron |
| Huijskens | Elisabeth G.W. |
| de Jong | Eefje |
| Koeleman | Hans |
| Tjeerdsma-de Bruin | Gerjanne |
| Kampinga | Greetje A. |
| Nijssen | Saskia |
| Ridwan | Ben |
